# Sleep Efficiency and Total Sleep Time in Individuals with Type 2 Diabetes with and without Insomnia Symptoms

**DOI:** 10.1155/2020/5950375

**Published:** 2020-07-17

**Authors:** Mohammed M. Alshehri, Abdulaziz A. Alkathiry, Aqeel M. Alenazi, Shaima A. Alothman, Jason L. Rucker, Milind A. Phadnis, John M. Miles, Patricia M. Kluding, Catherine F. Siengsukon

**Affiliations:** ^1^Physical Therapy and Rehabilitation Science Department, University of Kansas Medical Center, Kansas City, Kansas, USA; ^2^Physical Therapy Department, Jazan University, Jazan City, Southern Region, Saudi Arabia; ^3^Physical Therapy Department, Majmaah University, Almajmaah, Central Region, Saudi Arabia; ^4^Physical Therapy Department, Prince Sattam Bin Abdulaziz University, Alkharj City, Central Region, Saudi Arabia; ^5^Department of Biostatistics, University of Kansas Medical Center, Kansas City, Kansas, USA; ^6^Endocrinology Department, University of Kansas Medical Center, Kansas City, Kansas, USA

## Abstract

There is increasing awareness of the high prevalence of insomnia symptoms in individuals with type 2 diabetes (T2D). Past studies have established the importance of measuring sleep parameters using measures of central tendency and variability. Additionally, subjective and objective methods involve different constructs due to the discrepancies between the two approaches. Therefore, this study is aimed at comparing the averages of sleep parameters in individuals with T2D with and without insomnia symptoms and comparing the variability of sleep parameters in these individuals. This study assessed the between-group differences in the averages and variability of sleep efficiency (SE) and total sleep time (TST) of 59 participants with T2D with and without insomnia symptoms. Actigraph measurements and sleep diaries were used to assess sleep parameter averages and variabilities calculated by the coefficient of variation across 7 nights. Mann–Whitney *U* tests were utilized to compare group differences in the outcomes. Validated instruments were used to assess the symptoms of depression, anxiety, and pain as covariates. Objective SE was found to be statistically lower on average (85.98 ± 4.29) and highly variable (5.88 ± 2.57) for patients with T2D and insomnia symptoms than in those with T2D only (90.23 ± 6.44 and 3.82 ± 2.05, respectively). The subjective average and variability of SE were also worse in patients with T2D and insomnia symptoms, with symptoms of depression, anxiety, and pain potentially playing a role in this difference. TST did not significantly differ between the groups on averages or in variability even after controlling for age and symptoms of depression, anxiety, and pain. Future studies are needed to investigate the underlying mechanisms of worse averages and variability of SE in individuals with T2D and insomnia symptoms. Additionally, prompting the associated risk factors of insomnia symptoms in individuals with T2D might be warranted.

## 1. Introduction

Sleep disturbances, especially insomnia, are commonly reported by individuals with type 2 diabetes (T2D) [1, 2]. The association between insomnia symptoms and T2D may reflect possible bidirectional relationships through the dysregulation of the hypothalamic-pituitary-adrenal (HPA) axis, appetite-controlling hormones, and glucose metabolism. A preliminary study showed that individuals with insomnia have increased activation of the HPA axis, resulting in high cortisol production [3]. Increasing the cortisol level during poor night sleep is associated with glucose production from the liver [4]. Another possible mechanism was a U-shape association between short and long sleep duration in individuals with poor glycemic control [5]. As the mechanisms underlying this association remain unclear, it is important to determine the additive effect of insomnia symptoms on sleep parameters in individuals with T2D.

High night-to-night sleep variability, defined as irregular night-to-night sleep schedules due to misalignment between sleep-wake timing and the circadian system [6], is a common source of distress in individuals with chronic insomnia [7, 8]. For example, individuals with insomnia often compensate for a night of insufficient sleep by attempting to sleep longer the following night. While this may lead to a temporary improvement in sleep efficiency (SE) [9, 10], such detrimental sleep behavior may imbalance the regular sleep drive and cause difficulty falling asleep, impairing sleep duration and SE on subsequent nights [9, 10]. Sleep duration and SE are important sleep parameters reflecting overall sleep quality, which may be affected by variation in these sleep parameters [11, 12]. Although night-to-night sleep variability is common in contemporary life because of increasing economic and social demands [13] and a previous study showed that insomnia severity is not correlated with sleep variability [14], there is lack of evidence regarding night-to-night sleep variability in individuals with T2D and insomnia symptoms.

The averages and variabilities of sleep measures are indicators of an individual's sleep-wake rhythm [15, 16]; using both values could help provide a more thorough interpretation of sleep behavior [15–17]. In general, low variability and high average SE would reflect better quality of sleep. However, low SE variability does not represent poor or normal sleep quality in all cases. For instance, a person with poor or normal SE in all 7 nights of a week will show low variation in the measured SE, as their SE will be consistently poor or normal. Therefore, understanding the association between both the average and variability of sleep parameters may provide clear insight into current research for individuals with T2D [18].

Subjective and objective measures of sleep are important for assessing insomnia symptoms in clinical and research applications. Sleep diaries and actigraphy are useful tools to subjectively and objectively measure sleep parameters, respectively, including SE and total sleep time (TST) [19]. Due to the discrepancies between subjective and objective measures, simultaneously capturing both types of measurements may be needed to measure sleep parameters [20]. Sleep diaries measure sleep parameters based on the individual's best estimation, which may be affected by recall bias, whereas actigraphy is less sensitive in detecting the time taken for falling asleep and fails to detect waking after sleep onset [19]. In addition, depression symptoms might play a role in the subjective sleep measures for individuals with insomnia [21]. Despite these limitations, both measures are reliable in assessing the sleep-wake pattern across multiple nights and provide an indication of the response of TST and SE to treatment, which is key to empirically capturing sleep variability [22].

Shared health issues commonly present in individuals with T2D with sleep disturbances include sleep apnea, depression, anxiety, and pain [23–25]. Previous studies have shown the negative impact of these health conditions on sleep parameters (average and variability) [10, 26]. Older adults with insomnia have high variation in sleep and report morning pain, possibly because of depression and anxiety symptoms [27]. In a multiethnic study, the severity of sleep apnea episodes was found to be associated with greater sleep fragmentation and high variation in sleep duration was associated with stress and depression [28]. It has been suggested to consider accounting for the severity of depression and anxiety symptoms in sleep variability studies [20]. Given the high prevalence of insomnia symptoms in individuals with T2D, it is imperative to consider the shared health issues in this population to develop preventive strategies and treatment options.

Persistent sleep variability may result in poor sleep quality in individuals with T2D [29]. In addition, various T2D symptoms may explain the variation in sleep parameters including frequent nocturnal urination, hyperglycemia-related food consumption, hypoglycemia symptoms, obesity, pain, and distress [30]. A recent study showed that diabetes-related symptoms including neuropathic pain, diabetes distress, and depressive symptoms were more likely associated with sleep disturbances [31]. However, it is important to elucidate the additive effects of insomnia symptoms on sleep parameters in individuals with T2D. The purpose of this study was to compare the averages of sleep parameters in individuals with T2D with and without insomnia symptoms and to compare the variability of sleep parameters in this population. Secondarily, we assessed the association between the average and variability of sleep parameters in individuals with T2D with and without insomnia symptoms.

## 2. Materials and Methods

### 2.1. Design and Participants

This cross-sectional study enrolled 60 participants with self-reported T2D. Part of this project, which compared diabetes outcomes, was published elsewhere [32]. We used several recruitment sources between November 10, 2018, and April 15, 2019, including a research participant registry, a diabetes clinic, advertisements at a university research center, and flyers distributed in the community. The study was approved by the institutional review board of the University of Kansas Medical Center (IRB # STUDY00142985). Written informed consent was obtained from all participants during the first study visit.

### 2.2. Screening Procedure

All participants underwent phone and in-person screening sessions to ensure that they met the eligibility criteria. Individuals were included if they (1) self-reported T2D, which was confirmed by reviewing the participants' medication lists during the in-person screening session, (2) were 40–75 years old, (3) were able to understand and follow verbal commands in English, and (4) were able to attend the study visits and complete the testing procedures. Individuals were excluded if they (1) were at risk of untreated sleep apnea or restless leg syndrome using the STOP-Bang and restless leg syndrome diagnostic algorithm, respectively; (2) reported being pregnant; (3) reported consuming ≥15 drinks/week for men and ≥8 drinks/week for women; (4) self-reported a diagnosis of neurological diseases, bipolar disorder, seizure disorder, chronic fatigue syndrome, or rheumatic diseases, being on dialysis, blindness, or transfemoral amputation; (5) reported performing shift work; (6) scored ≥7 out of 10 on the Brief Pain Inventory; (7) scored ≥21 on the Beck Depression Inventory; and (8) scored ≥15 on the Generalized Anxiety Disorder 7-item scale. The exclusion criteria were established to minimize the external influence of common health issues on sleep quality in individuals with T2D.

### 2.3. Group Allocation

All participants were stratified based on the Insomnia Severity Index. Participants who scored >10 on the Insomnia Severity Index were included in the T2D and insomnia group (T2D+Insomnia), while participants who scored ≤10 were included in the T2D-only group (T2D only). Including only patients with insomnia symptoms above the cutoff score of 10 provided optimal sensitivity (97.2%) and specificity (100%) for the detection of insomnia in a clinical sample [33]. Additionally, we confirmed that the self-reported symptoms of difficulty falling asleep, maintaining sleep, or waking up too early were present for at least 3 nights/week for the past 3 months in the participants in the T2D+Insomnia group. Previous research showed that the Insomnia Severity Index does not correlate with sleep variability [14].

### 2.4. Measures

#### 2.4.1. Objective Sleep Parameters

SE and TST were tested using an actigraph device (Model wGT3X-BT, ActiGraph Corp., Pensacola, FL) as previously described [19]. The actigraph is a three-axis accelerometer, which has been validated for use with individuals with insomnia. It objectively differentiates individuals with poor sleep quality from those with good sleep quality [34]. The actigraph is a small, noninvasive device that records limb movements using electrical impulses at 30–100 Hz. Participants were instructed to wear the actigraph on their nondominant wrist all day for 8 consecutive days and 7 nights, including weekend nights, to capture habitual sleep patterns. The participants were requested to temporarily remove the device if submerged in water for more than 30 minutes (i.e., bathing or swimming). A blinded trained assessor scored the actigraph data using the ActiLife software (version 6.11.8, ActiGraph Corp.). Using the Cole–Kripke algorithm, which has been validated for adult populations [35], sleep parameters such as SE and TST were objectively assessed. Additionally, the blinded assessor used the sleep diary to obtain a better estimation of the time in bed and time out of bed and removed invalid sleep periods with reference to the actigraphy data. Invalid wear time was defined as wearing the actigraph device for less than 400 minutes per day. A study showed the limited agreement between actigraph (Model wGT3X) and sleep diaries. They found that the actigraph and sleep diary differed by −11.3 min for TST and by −0.2% for SE in people with T2D [36].

#### 2.4.2. Subjective Sleep Parameters

Participants were asked to complete the Consensus Sleep Diary to provide the best estimation of time in bed, time out of bed, duration of sleep latency, waking after sleep onset, and early morning awaking [37]. The provided information on the sleep diary was used to calculate the TST and SE. The TST was calculated as total time in bed − the duration of sleep latency − waking after sleep onset − early morning awaking. Then, the SE was calculated as (TST/total time in bed) × 100.

#### 2.4.3. Possible Covariates

Information on age, sex, body mass index, education, and ethnicity was collected at the assessment session. Current *positive* airway pressure (PAP) machine usage and the severity of symptoms of pain, depression, and anxiety were measured. Participants were asked if they were using a PAP machine with a yes/no question (“Do you use any type of PAP machine?”). The PAP machine is commonly prescribed for individuals with T2D and obstructive sleep apnea to help reduce the number of nocturnal apnea and hypopnea episodes [38]. Pain severity symptoms were measured using the Brief Pain Inventory, which is a validated and reliable measure commonly used to assess pain in diabetic peripheral neuropathy [39]. High Brief Pain Inventory scores indicate severe pain symptoms. Depression symptoms were measured using the Beck Depression Inventory, which has been shown to have high reliability and good validity. The Beck Depression Inventory consists of 21 self-reported items rated on a three-point Likert scale, with scores ≥ 21 indicating severe depression symptoms [40]. The Generalized Anxiety Scale contains seven items adding up to a total score ranging from 0 to 21, with higher scores indicating severe anxiety symptoms. The Generalized Anxiety Scale has been shown to be highly sensitive and specific for the detection of anxiety symptoms, and its scores correlate with those of other anxiety scales [41].

### 2.5. Power Analysis

All sample size calculations were performed using the PASS 14.0 software with a linear mixed model for continuous outcomes. Based on a previous study that investigated sleep variability in individuals with chronic insomnia, Cohen's *d* effect sizes for sleep latency, TST, and SE were 0.59, 0.71, and 0.78, respectively [42]. Our sample size calculations were conservatively based on the minimum of the above-mentioned effect sizes. Specifically, we selected Cohen's *d* of 0.59, corresponding to a change of ±2/3 SD from the mean [43]. To account for the possibility of a correlation between seven repeated measurements (corresponding to 7 nights of sleep), we used a mixed model with a compound symmetry covariance structure (correlation of 0.5 between any pair of measurements) to conduct the sample size calculations. The results of these calculations indicated that 28 participants in each group were needed to detect a significant difference in sleep variability between groups at the 0.05 significance level with a power of 0.85.

### 2.6. Data Synthesis and Statistical Analysis

All data analyses were performed using SPSS 25.0 for Mac (IBM Corp., Armonk, NY). Descriptive statistics included means and standard deviations for continuous variables, while frequencies were used to describe categorical variables. Descriptive measures of time in bed with mean SE were established as visual circular data using the R statistical package (R Foundation for Statistical Computing, Vienna, Austria). All sleep parameters (TST and SE) are presented as averages and variabilities of 7 nights. The coefficient of variation was calculated using the following equation: Coefficient of variation = (standard deviation/mean) × 100% to analyze within-subject variability of nighttime sleep across 7 nights. This calculation provides a percentage value, with higher numbers suggesting higher sleep variability [44].

For demographics and clinical variables, chi-square and independent sample *t*-test analyses were used to assess differences between groups in categorical and continuous variables, respectively. For the main analysis, Mann–Whitney *U* tests were utilized to compare group differences in the average and variability of sleep parameters (SE and TST). In performing the exploratory analyses, complex relationship between insomnia and T2D might be necessary to control for several factors by adjusting for covariates to investigate the differences between groups in the sleep parameters. However, due to the small sample size and the fact that the covariates were not included in the power calculation, these complex relationships can only be investigated in an exploratory manner. We decided to use exploratory analysis because the groups significantly differed in age and depression, anxiety, and pain symptoms. Thus, to control for age and symptoms of depression, anxiety, and pain, a multivariable general linear model was used to examine the differences between groups in sleep parameter averages and sleep variability. The group (*β* = T2D + Insomnia group − T2D‐only group) was included as an independent variable with sleep parameters as dependent variables. For the secondary aim, multiple linear regression tests and scatterplots were utilized to assess the association between averages and variability values for both groups. All tests were conducted at an alpha level of 0.05.

## 3. Results

### 3.1. Descriptives

**Figure 1:**
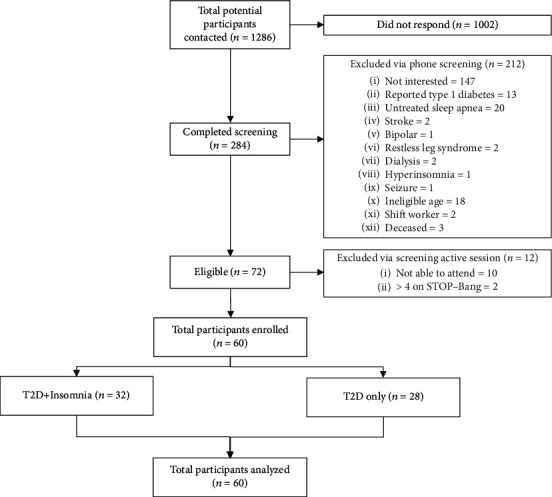
Participant recruitment process.

**Figure 2:**
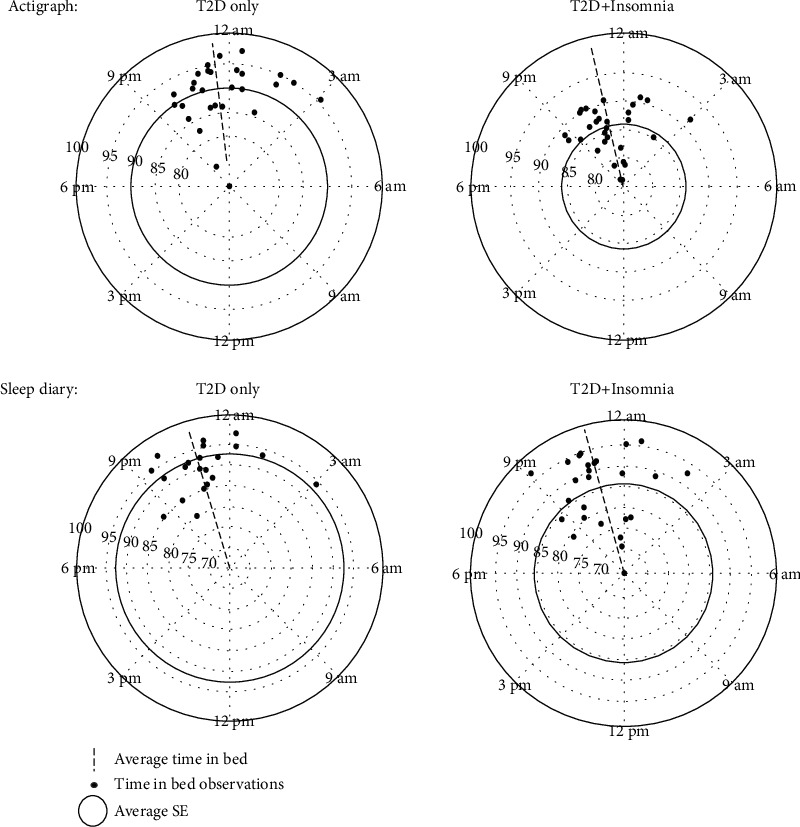
Descriptive means of time in bed distribution with sleep efficiency as a magnitude for both groups using actigraph and sleep diary. SE = sleep efficiency; TST = total sleep time.

Fifty-nine participants were recruited and their data included in the final analysis. The participant flow diagram is shown in Figure 1. The demographics and clinical variables of the participants in both groups are summarized in Table 1. The participants in both groups were similar in all demographics except age (*p* = 0.02). The mean Insomnia Severity Index score was 4.64 ± 3.15 in the T2D-only group and 16.00 ± 3.08 in the T2D+Insomnia group (*p* < 0.001). Participants in the T2D+Insomnia group reported more severe symptoms of depression (11.00 ± 5.91) and anxiety (7.41 ± 4.71) than those in the T2D-only group (4.79 ± 4.77 and 2.93 ± 4.00, *p* < 0.001, respectively). The severity of pain was significantly higher in the participants in the T2D+Insomnia group (3.27 ± 2.10) than in those in the T2D-only group (1.55 ± 1.67). The distribution of subjective and objective measures of time in bed with mean SE as the magnitude in both groups is shown in Figure 2.

### 3.2. Differences in Sleep Parameters between Groups

As shown in Table 2, the participants in the T2D+Insomnia group had significantly lower averages of objective SE (85.98 ± 4.29) than those in the T2D-only group (90.23 ± 6.44). Regarding the subjective measures, the participants in the T2D+Insomnia group had lower averages of SE (85.75 ± 8.70) than those in the T2D-only group (92.61 ± 5.33). Regarding TST, the subjective average was lower in the T2D+Insomnia group (420.04 ± 72.34) than in the T2D-only group (474.52 ± 71.43), while no significant between-group difference was observed in the objective TST average.

The objective measures showed significantly higher variability in SE in the T2D+Insomnia group (5.88 ± 2.57) than in the T2D-only group (3.82 ± 2.05). Regarding the subjective measures, the participants in the T2D+Insomnia group had higher variability in SE (11.13 ± 9.02) than those in the T2D-only group (4.99 ± 4.63). Regarding TST, subjective variability was higher in the T2D+Insomnia group (22.71 ± 16.59) than in the T2D-only group (13.43 ± 8.03), while no significant between-group difference was observed in objective TST variability.

### 3.3. Multivariable General Linear Model

Table 3 presents the between-group differences in the objective averages of sleep parameters after controlling for covariates. After controlling for age and pain, depression, and anxiety severities, the objective measures showed significantly lower averages of SE (*β* = −4.63, *p* = 0.01) in the T2D+Insomnia group than in the T2D-only group. However, the between-group differences in the averages of objective TST were not significant when age and pain, depression, and anxiety severities were incorporated in the model.

Table 3 presents the between-group differences in subjective averages of sleep parameters after controlling for covariates. Subjective measure showed significant lower averages of SE (*β* = −6.43, *p* = 0.008) in the T2D+Insomnia group than in the T2D-only group when only adding age as a covariate. There were no significant group differences in the subjective averages of SE after controlling for pain, depression, and anxiety severities. In addition, no significant group differences were observed in the averages of subjective TST after controlling for all covariates.

A significant between-group difference was observed in the objective variability of SE (*β* = 1.98, *p* = 0.03) when controlling for age and pain severity (Table 3). The participants in the T2D+Insomnia group showed higher variability of objective SE than those in the T2D-only group. The participants in the T2D+Insomnia group showed significant increase in variability of subjective SE (*β* = 4.93, *p* = 0.04) after controlling for age only. Controlling for age and pain, depression, and anxiety severities yielded no significant between-group differences in variability in all sleep parameters using objective or subjective measures.

### 3.4. Multiple Linear Regression

**Figure 3:**
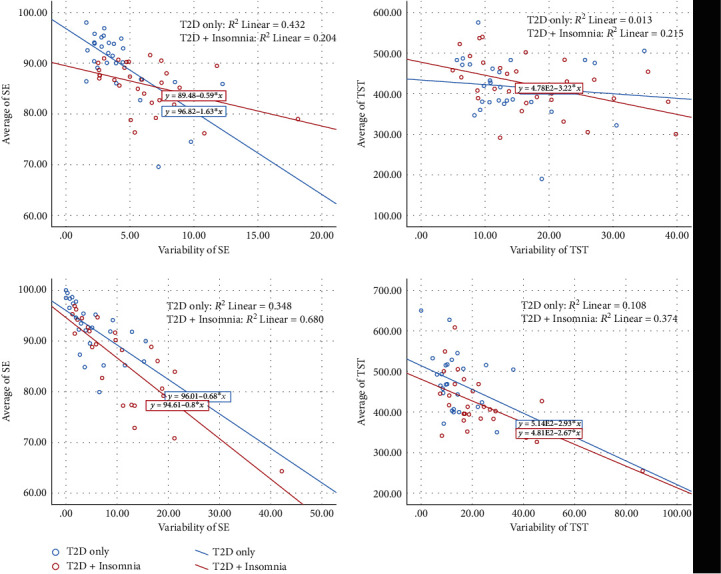
Graphs to visualize the relationship between sleep variability and average of SE and TST for both groups using actigraph and sleep diary. SE = sleep efficiency; TST = total sleep time; T2D = type 2 diabetes.

As shown in Figure 3, a significant relationship between the average and variability of objective SE (*R*^2^ = 0.20, *p* = 0.01) was found in the T2D+Insomnia group. In addition, a significant relationship between the average and variability of objective TST was found in the T2D+Insomnia group (*R*^2^ = 0.21, *p* = 0.009). Regarding the subjective measures, significant relationships between the average and variability of SE (*R*^2^ = 0.68, *p* < 0.001) and TST (*R*^2^ = 0.37, *p* = 0.001) were found in the T2D+Insomnia group.

There were significant relationships between the average and variability in objective SE (*R*^2^ = 0.43, *p* < 0.001) and in subjective SE (*R*^2^ = 0.35, *p* = 0.002) in the T2D-only group. There were no significant relationships between the average and variability in objective TST or the average and variability in subjective TST.

## 4. Discussion

The aim of this study was to compare the averages and variabilities of the sleep parameters, SE and TST, in individuals with T2D with and without insomnia symptoms using objective and subjective measurements. Among the key findings of our study was the detection of lower averages and higher variability of SE in the participants with T2D and insomnia symptoms compared to those with T2D only using both subjective and objective measures. Psychological symptoms, including pain, depression, and anxiety, were found to play a role in the differences between groups in the variability of SE using both subjective and objective measures. Additionally, the subjective measures showed that the participants with T2D and insomnia symptoms had lower average of TST and higher variability in TST than those with T2D only. No differences in these measures were observed after controlling for the age of the participants. The subjective and objective sleep measures suggested that patients with T2D and insomnia symptoms exhibit worse sleep parameter averages compared to patients with T2D only.

Understanding the variability in sleep parameters through analysis of the average scores may provide important insight into the sleep patterns of individuals with T2D and insomnia symptoms. Consistent with the findings of our work, a study by Buysse et al. with older adults with insomnia showed high variability in SE and TST assessed using subjective approaches, but not in TST measured objectively, possibly reflecting the fact that individuals with insomnia commonly underestimate sleep duration [42]. Additionally, the authors reported worse average scores in subjective, but not in objective, measures of sleep parameters. Possibly, more nights of measurements may introduce long-term variability in sleep parameters as suggested by previous studies [42, 45]. A recent study showed that at least 5 nights for SE and more than 7 nights for TST were needed for reliable actigraphy measurements. However, subjective measurements required at least 6 nights to be considered reliable [46]. Alternatively, individuals with T2D may have misperceptions regarding their sleep, which may increase the variation in subjectively quantified sleep parameters. Previous studies have argued that individuals with T2D had poor SE compared to individuals without T2D [47, 48]. Our study provided evidence that insomnia severity is an important factor that should be considered when measuring the sleep quality of individuals with T2D given the high prevalence of sleep disturbances in this population. Buysse et al. showed no difference between groups in the number of comorbidities, which may explain the consistency of their findings with our results regarding the average values and SE variability [42]. However, our study targeted a population with T2D who commonly reported several sleep disturbances, such as nocturia, which may have increased the variability in sleep parameters. Our results contrast those of a multiethnic study that found higher correlations between self-reported sleep duration and actigraph measures [49]. Interestingly, measures of variability in sleep parameters, except for subjective measures of SE and TST, failed to show differences between participants with and without clinical insomnia [49]. Possible reasons for the inconsistency in the findings include recall bias and imprecision of self-reported sleep duration. Additionally, the authors measured SE and TST over 4 to 5 nights, which may also explain the difference in the findings.

Our study supports the importance of measuring both the variations and averages of sleep parameters. For example, we found no differences between groups in the variability of TST measured using the actigraph. However, we found differences in mean scores, which may explain the consistently worse sleep outcomes across most of the measured nights of sleep in individuals with T2D and insomnia symptoms. Overall, SE variability is higher in individuals with T2D and insomnia symptoms, which may explain the inconsistency of poor SE across all measured nights. This may suggest that several nights of sleep recovery are needed for individuals with insomnia symptoms to compensate for the sleep deficit accumulated over previous nights. Additionally, whether measured using subjective or objective approaches, higher averages of SE and TST were associated with lower variability in SE and TST in participants with and without insomnia symptoms. These findings indicate the importance of improving SE and TST to decrease the variation in sleep patterns in individuals with T2D.

Psychological domains, aging, and advanced sleep technology may explain the different observations between objective and subjective sleep measures. A previous study found discrepancies between subjective and objective sleep measurements due to psychological factors and actigraph sensitivity [50]. Discrepancy between objective and subjective measures has been reported by several studies as summarized in a recent review [20]. In this summary of sleep studies, the authors noted that failure to control for potential confounders was a main limitation in sleep research [20]. We found no differences between groups in averages and variability of subjective TST after controlling for covariates. Under- and overestimations of sleep duration in subjective reporting are influenced by objective sleep quality and psychological factors affecting individuals with sleep disorders [50]. While our power calculations did not account for covariates, which should be considered in future research, no changes were observed in the average of SE in objective measures after controlling for age and symptoms of pain, depression, or anxiety. These factors are commonly associated with both T2D and insomnia, with the additive effect of insomnia symptoms resulting in worse sleep parameters.

Our findings showed that psychological symptoms, including those of depression and anxiety, play a role in SE variability measured using objective and subjective approaches. We found that both subjective and objective variability in SE was no longer significantly different between groups when the statistical model incorporated psychological symptoms, but not when controlled for age or pain severity. Previously published work found that subjective SE corresponds to its objective assessment and is influenced by psychosocial factors [51]. This may explain the worse subjectively measured SE in patients with T2D and insomnia symptoms observed even after controlling for age. However, when adding severity of symptoms of pain, depression, or anxiety, no differences were noted between groups. No other significant differences were found in the variabilities of SL and TST measured objectively after controlling for covariates. T2D risk factors, such as age, hyperglycemia, and depression, contribute by increasing sleep disturbances, which may explain the lack of differences between groups in the averages and variabilities of SE and TST. Despite the known greater variability of sleep in individuals with T2D compared to healthy participants, further research is needed to investigate the complex relationship between psychological factors and variability in sleep parameters in individuals with T2D and insomnia symptoms.

Several limitations of this study need to be considered in the interpretation of the findings and in developing future research. Although this study was powered based on the published characteristics of the general population, there is a need to modify the calculations for the diabetic population by taking into account common external variables. There is currently no recommendation on the optimal number of nights that should be assessed to measure sleep parameters in individuals with T2D and insomnia symptoms. We measured the variability across 7 nights of sleep, but a different presentation would be expected if more nights were included to capture habitual sleep patterns. Greater night-to-night variability was previously shown to require more measured nights to accurately estimate sleep quality [10, 52]. We were not able to determine normal sleep variability in the T2D population, which would have been helpful in guiding future work to identify a cutoff score of sleep variability that would predict poor sleep quality. Despite its high sensitivity and specificity, the Insomnia Severity Index does not correlate with sleep variability [14], suggesting that using insomnia diagnostic criteria may result in inconsistent results. Although this study focused on individuals with T2D, including healthy participants as a third group would help in distinguishing the sleep patterns in the T2D population. Screening for sleep apnea using gold standard measurements such as polysomnography may help in excluding confounding variables that might add further variation to the sleep parameters. Age plays a more important role than sex in sleep research [53]. Previous studies have reported that older adults have poorer sleep quality and lower slow wave sleep than younger ones [54, 55]. Although we controlled for age in our study, an age-matched group should be included in future studies. Finally, our study did not control for concomitant medications (types or numbers), and we recommend that future studies should collect a medication list due to the potential effect of medications on sleep variability.

## 5. Conclusion

This study observed high SE variability and poor SE in individuals with T2D and insomnia symptoms compared to those with T2D only, with the statistical analyses suggesting that psychological symptoms may explain the observed differences. There were different observations between objective and subjective measurements of sleep duration, which may reflect the nature of actigraph and sleep diary measurements. Individuals with T2D and insomnia symptoms had worse symptoms of depression, anxiety, and pain than those without insomnia symptoms. Our findings indicate that further research is warranted to investigate the complex relationship between the variability in sleep and psychological factors in individuals with T2D and insomnia symptoms. In addition, using SE variability rather than TST variability might provide important methodological means to investigate the association between night-to-night sleep variability and diabetes outcomes for future studies in individuals with T2D and insomnia symptoms. A longitudinal design may help elucidate the impact of sleep variation on the psychological domains and diabetes outcomes of individuals with T2D.

## Figures and Tables

**Table 1:** Comparison of demographics and clinical variables between participants with T2D with and without insomnia symptoms using the independent sample *t*-test and chi-square test.

	T2D only (mean ± SD)	T2D+Insomnia (mean ± SD)	*p* value
Age	64.79 ± 6.50	60.28 ± 7.83	0.02^a^
Sex, female, *n* (%)	13 (46.42)	19 (59.37)	0.44^b^
Body mass index	35.57 ± 7.90	32.54 ± 5.26	0.08^a^
Education, *n* (%)			0.42^b^
Eight grades or fewer	0 (0)	1 (3.12)	
High school	5 (17.85)	6 (18.75)	
Some college	11 (39.28)	6 (18.75)	
College graduate	7 (25)	11 (34.37)	
Graduate degree	5 (17.85)	8 (25)	
Ethnicity, *n* (%)			0.28^b^
White	21 (75)	23 (71.87)	
Black	5 (17.85)	3 (9.37)	
Other	2 (7.14)	6 (18.75)	
Insomnia Severity Index	4.64 ± 3.15	16.00 ± 3.08	<0.001^a^
Brief Pain Inventory (score)	1.55 ± 1.67	3.27 ± 2.10	0.001^a^
Beck Depression Inventory (score)	4.79 ± 4.77	11.00 ± 5.91	<0.001^a^
Generalized Anxiety Scale (score)	2.93 ± 4.00	7.41 ± 4.71	<0.001^a^
Using positive airway pressure, *n* (%)			0.74^b^
Never	18 (64.28)	20 (62.5)	
Current	9 (32.14)	12 (37.5)	

^a^Independent sample *t-*test; ^b^chi-square test.

**Table 2:** Comparison of sleep variabilities and averages in SE and TST between the participants with T2D with and without insomnia symptoms using the Mann–Whitney *U* test.

		T2D only (mean ± SD)	T2D+Insomnia (mean ± SD)	*p* value
Average of	SE (%)	Actigraph		
		90.23 ± 6.44	85.98 ± 4.29	0.005
	TST (min)	415.22 ± 73.70	425.28 ± 63.03	0.58

Coefficient of Variation of	SE	3.82 ± 2.05	5.88 ± 2.57	0.002
	TST	13.77 ± 7.21	17.18 ± 9.47	0.13

Average of		Sleep diary		
	SE (%)	92.61 ± 5.33	85.75 ± 8.70	0.001
	TST (min)	474.52 ± 71.43	420.04 ± 72.34	0.006

Coefficient of Variation of	SE	4.99 ± 4.63	11.13 ± 9.02	0.002
	TST	13.43 ± 8.03	22.71 ± 16.59	0.004

T2D: type 2 diabetes; SE: sleep efficiency; TST: total sleep time.

**Table 3:** Multivariable general linear model used to examine the differences between groups in objective and subjective means of sleep parameters and sleep parameter variability.

		Model	*R* ^2^	Adjusted *R*^2^	*β*	*p* value
Actigraph						
Average of	SE (%)	1	0.14	0.11	-4.31	0.005
		2	0.14	0.09	-4.47	0.008
		3	0.14	0.06	-4.63	0.01
	TST (min)	1	0.001	-0.03	3.62	0.85
		2	0.02	-0.02	13.05	0.53
		3	0.04	-0.05	21.49	0.35
Coefficient of Variation of	SE	1	0.11	0.08	2.19	0.01
		2	0.12	0.07	1.98	0.03
		3	0.12	0.04	1.87	0.06
	TST	1	0.03	0.003	3.15	0.18
		2	0.05	0.008	2.01	0.43
		3	0.21	0.14	0.14	0.95

Sleep dairy						
Average of	SE (%)	1	0.15	0.11	-6.43	0.008
		2	0.21	0.15	-4.59	0.07
		3	0.27	0.18	-3.06	0.25
	TST (min)	1	0.13	0.09	-35.69	0.11
		2	0.14	0.08	-29.18	0.23
		3	0.17	0.08	-17.70	0.49
Coefficient of Variation of	SE	1	0.13	0.10	4.93	0.04
		2	0.18	0.13	3.19	0.21
		3	0.26	0.17	1.39	0.60
	TST	1	0.19	0.15	4.92	0.24
		2	0.19	0.14	4.22	0.37
		3	0.21	0.12	3.11	0.53

Model 1: controlling for age. Model 2: controlling for age and pain severity. Model 3: controlling for age and pain, depression, and anxiety severities; T2D-only as the reference group. SE = sleep efficiency; TST = total sleep time.

## Data Availability

The corresponding author can make data available on request through a data access committee from University of Kansas Medical Center institutional review board via IRBhelp@kumc.edu.
